# Oxaliplatin-induced Pulmonary Toxicity: A Rare but Serious Complication

**DOI:** 10.7759/cureus.7483

**Published:** 2020-03-31

**Authors:** Krishna H Suthar, Salwan Al Mutar, Rohit Venkatesan

**Affiliations:** 1 Internal Medicine, University of Texas Medical Branch, Galveston, USA; 2 Medical Oncology, Ohio State University, Columbus, USA; 3 Oncology, University of Texas MD Anderson Cancer Center, Galveston, USA

**Keywords:** oxaliplatin, pulmonary toxicity, chemotherapy-related toxicity, pneumonitis, folfox

## Abstract

The FOLFOX regimen (oxaliplatin, leucovorin, and 5-fluorouracil) is FDA approved for use in patients with colorectal cancer and other gastrointestinal malignancies. The initial phase III randomized controlled trials that led to FDA approval of oxaliplatin with leucovorin and 5-fluorouracil showed a less than 1% incidence of pulmonary fibrosis and grade IV pulmonary toxicities. Here we describe two cases of pulmonary toxicity in patients with metastatic colorectal cancer treated with FOLFOX and briefly review the literature regarding oxaliplatin-induced pulmonary toxicity.

## Introduction

Oxaliplatin is a third-generation platinum derivative that exerts cytotoxic effects by interrupting DNA synthesis [1]. Oxaliplatin-containing regimen is used as a standard treatment for adjuvant therapy in patients with advanced stage gastrointestinal malignancies [2,3]. The toxicity profile of oxaliplatin, established in preclinical and clinical studies, consists of peripheral sensory neuropathy, gastrointestinal and liver toxicity, thrombocytopenia, and hypersensitivity reactions [4]. However, various case reports have been published describing oxaliplatin-associated acute interstitial lung disease and pulmonary fibrosis [5-12]. Here we describe two such cases of pulmonary toxicity in patients with metastatic colorectal cancer treated with FOLFOX.

## Case presentation

Case 1

A 62-year-old female with a 10-pack year history of tobacco use disorder (quit 10 years prior to presentation) presented with abdominal pain and vaginal bleeding. Initial workup was concerning for malignancy. Colonoscopy was attempted, but the colonoscope could not be advanced beyond the rectosigmoid junction due to stricture and suspected mass lesion 17 cm from the anal verge. Biopsy of the mass revealed adenocarcinoma of the gastrointestinal tract (*KRAS* mutated, *NRAS* and *BRAF* wild type, microsatellite stable), and the initial stage was IV. The patient completed 12 doses of mFOLFOX-6 with clinical response. Repeat imaging demonstrated new ground-glass pulmonary opacities, but the patient was asymptomatic so chemotherapy was continued without dosage adjustments.

**Figure 1:**
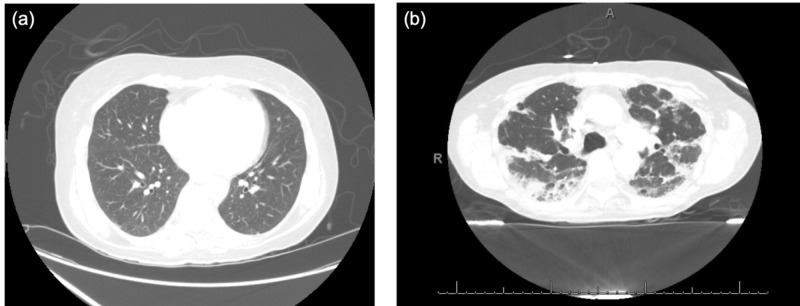
High-resolution computed tomography of the chest for patient 1. (a) High-resolution computed tomography of the chest for patient 1 prior to the initiation of mFOLFOX-6, (b) High-resolution computed tomography of the chest for patient 1 after 15 cycles of mFOLFOX-6 demonstrates extensive mixed solid and ground-glass opacities with mild bronchiectasis.

After her 15th dose, she presented to the hospital with progressive dyspnea, a dry cough, and hypoxia. The CT chest revealed worsening of her known bilateral ground-glass opacities with mild bronchiectasis and bilateral lower lobe segmental and subsegmental pulmonary emboli (Figure 1). Anticoagulation was started for the venous thromboemboli. Infectious workup was unremarkable. Subsequent cycles of chemotherapy were held, and she was initiated on high-dose steroids and inhaled N-acetylcysteine (NAC). Bronchoscopy was deferred as the patient opted for hospice care due to evidence of disease progression. She was discharged with plans for a steroid taper. 

Case 2

A 38-year-old female with a history of alcohol use disorder, chronic pancreatitis, active 21-pack-year smoking history, and a recent episode of community acquired pneumonia presented to the hospital with abdominal pain, nausea, and chronic diarrhea. Imaging revealed peritoneal carcinomatosis, and omental biopsy showed metastatic adenocarcinoma. Colonoscopy confirmed the diagnosis of a well-differentiated colorectal adenocarcinoma with microsatellite instability. Initial imaging showed no evidence of significant pulmonary pathology. She was initiated on mFOLFOX-6 and bevacizumab [13]. However, after cycle 1 of chemotherapy, she presented with dyspnea, and CT of the thorax demonstrated a small right pleural effusion and pulmonary edema. Symptoms improved with diuretic therapy. 

**Figure 2:**
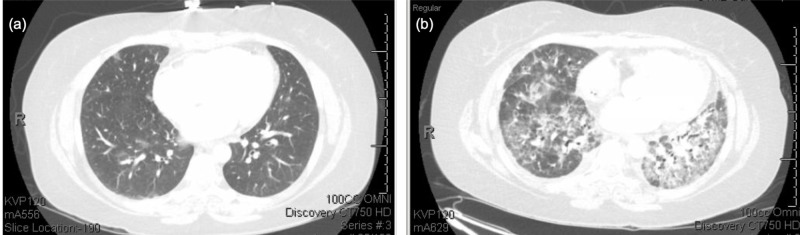
High-resolution computed tomography of the chest for patient 2. (a) High-resolution computed tomography of the chest for patient 2 prior to initiation of chemotherapy, (b) High-resolution computed tomography of the chest for patient 2 after three cycles of mFOLFOX + bevacizumab showing extensive bilateral ground-glass opacities.

She completed two more chemotherapy cycles but was hospitalized after cycle 3. Her clinical course was complicated by hospital acquired pneumonia, managed with vancomycin and cefepime. However, her respiratory status continued to worsen, requiring transfer to the medical ICU for respiratory failure. Antibiotics were broadened and steroid therapy was initiated. Repeat CT of the thorax showed worsened pulmonary edema with ground-glass opacifications and septal thickening likely secondary to pneumonitis (Figure 2). Bronchoscopy cultures and cytology were negative, but there was evidence of inflammatory infiltrate. Her respiratory condition improved while on prednisone 40 mg daily. Given symptom improvement, she went on to receive cycle 4 of mFOLFOX-6 without bevacizumab due to concerns for bevacizumab-induced pneumonitis. Shortly after completing the cycle, her respiratory status rapidly declined requiring intubation. At that time, the patient’s family decided to proceed with comfort care, and she expired shortly after extubation. 

## Discussion

Pulmonary toxicity is a known complication of various chemotherapy agents such as gefitinib, bleomycin, and mitomycin [14]. In the phase III randomized controlled trials that lead to FDA approval of FOLFOX, the incidence of reported pulmonary toxicity was <1%; however, in the last 10 years, there have been increasing numbers of cases of this adverse reaction reported in the literature with one case review reporting a 76.9% mortality rate despite conventional treatment with corticosteroids [15,16]. Oxaliplatin is the only known agent in the FOLFOX regimen to have pulmonary side effects as 5-fluorouracil and leucovorin have not been implicated in the development of pulmonary toxicities alone [8,16,17]. The presentation of oxaliplatin-induced pulmonary toxicity can range from early evidence of interstitial pneumonitis to severe pulmonary fibrosis. It is important to note that the diagnosis of drug-induced pneumonitis is one of exclusion and requires evaluation to exclude more common etiologies of respiratory symptoms such as infections, pulmonary embolism, lymphangitic carcinomatosis, heart failure, and pulmonary bleeding. 

The exact mechanism by which oxaliplatin leads to parenchymal lung disease is unknown. One proposed theory is that oxaliplatin has glutathione depleting effects. The loss of glutathione in the lungs can lead to an increased susceptibility of the lung parenchyma to oxidative damage [18]. Shimura et al. completed a retrospective study of 734 patients with colorectal cancer treated with FOLFOX or FOLFIRI (5-fluorouracil, leucovorin, and irinotecan) and reported that smoking, pulmonary metastasis, and the presence of a pre-existing pulmonary pathology were linked to the development of pulmonary toxicity [17]. However, given the published studies of oxaliplatin-induced pulmonary toxicity, it was difficult to truly draw conclusions regarding risk factors.

The cases presented here demonstrate a temporal relationship between FOLFOX therapy and development of both clinical symptoms and radiographic evidence of interstitial lung disease. In both cases, alternative etiologies for their respiratory symptoms were ruled out. The initial chemotherapy regimen of patient 2 included bevacizumab, which is also associated with pulmonary toxicity, but her respiratory function continued to decline after chemotherapy with only mFOLFOX-6. Thus, her case could represent pulmonary toxicity due to both bevacizumab and oxaliplatin or oxaliplatin alone. It is also important to note there is a difference in time from initiation of chemotherapy to symptom onset in both patients. Patient 1 tolerated 15 cycles of mFOLFOX-6 without radiographic or symptomatic pulmonary toxicity, while patient 2 received three cycles of mFOLFOX-6 and bevacizumab and one cycle of mFOLFOX-6 prior to the development of pulmonary toxicity. Factors that could account for this difference between the patients are as follows: (1) the use of bevacizumab in patient 2, and (2) patient 1 had a remote history of tobacco use while patient 2 actively smoked and had a recent pneumonia. This reflects the potential risk factors presented by Shimura et al. [17] . 

Currently, there are no established guidelines to treat oxaliplatin-induced pulmonary toxicity. If there is evidence of pulmonary toxicity, oxaliplatin therapy should be discontinued. A recent review of 45 cases of oxaliplatin-induced pulmonary toxicity demonstrated that patients with only mild respiratory symptoms had 100% regression in symptoms upon cessation of oxaliplatin [16]. However, further therapeutic modalities are indicated in more symptomatic patients. There has been a documented positive response to glucocorticoid therapy [19]. NAC is also used as it is believed to replete glutathione, the small molecule antioxidant that oxaliplatin is believed to deplete [18]. In our first case, the patient demonstrated improvement with glucocorticoid therapy and NAC. De Weerdt et al. proposed the use of immune-modulating agents such as intravenous immune globulins (IVIg) and cyclophosphamide as potential therapeutic options. IVIg is thought to reduce the deposition of excessive extracellular proteins, such as collagen-I, in the lung to ultimately limit progression of pulmonary fibrosis [16]. Cyclophosphamide, a nitrogen mustard-alkylating and lymphocyte-modulating agent, is a steroid-sparing agent used to reduce steroid therapy in patients with inflammatory disorders and associated interstitial lung disease [16]. Another potential therapy is imatinib, a c-Abl tyrosine kinase inhibitor. The normally functioning c-Abl tyrosine kinase promotes fibrosis through transforming growth factor β (TGF-β), a key factor in the development of fibrotic diseases. Imatinib inhibits this process resulting in reduced fibrosis. Studies thus far demonstrate that Imatinib has been successful in treating similar toxicities such as bleomycin-induced pulmonary fibrosis [20]. In their recent review of 45 published cases of oxaliplatin-induced pulmonary fibrosis, De Weerdt et al. found that oxaliplatin-induced pulmonary fibrosis can be fatal in respiratory insufficient patients with 76.9% of mechanically ventilated patients (10/13) passing away despite therapy [16]. Of those mechanically ventilated patients, 12 were treated only with monotherapy (high-dose steroids) and multiple modalities (IVIg and cyclophosphamide) were used in one patient, who survived. 

## Conclusions

Oxaliplatin-induced pulmonary toxicity is a rare complication of FOLFOX therapy, and data regarding risk factors and optimal therapy are limited. Larger analyses are needed to identify if there are any pre-existing conditions or risk factors that could increase a patient’s risk for pulmonary toxicity. Further studies are required to determine if there is a role for establishing baseline pulmonary function testing or high-resolution CT of the thorax prior to starting therapy along with active monitoring for signs and symptoms of intestinal lung disease in the management of these patients, especially if the patient has a pre-existing pulmonary pathology or history of smoking. Currently, glucocorticoids are used in the management of oxaliplatin-induced pulmonary disease, but further studies are needed to determine optimal regimens and explore the potential role of NAC, cyclophosphamide, and imatinib.
